# All Clinically-Relevant Blood Components Transmit Prion Disease following a Single Blood Transfusion: A Sheep Model of vCJD

**DOI:** 10.1371/journal.pone.0023169

**Published:** 2011-08-17

**Authors:** Sandra McCutcheon, Anthony Richard Alejo Blanco, E. Fiona Houston, Christopher de Wolf, Boon Chin Tan, Antony Smith, Martin H. Groschup, Nora Hunter, Valerie S. Hornsey, Ian R. MacGregor, Christopher V. Prowse, Marc Turner, Jean C. Manson

**Affiliations:** 1 The Roslin Institute and Royal (Dick) School of Veterinary Studies, University of Edinburgh, Roslin, Edinburgh, United Kingdom; 2 School of Veterinary Medicine, College of Medical, Veterinary and Life Sciences, The University of Glasgow, Glasgow, United Kingdom; 3 The Institute for Animal Health, Compton, Berkshire, United Kingdom; 4 Institute for Novel and Emerging Infectious Diseases, Friedrich-Loeffler-Institut, Federal Research Institute for Animal Health, Germany; 5 National Science Laboratory, Scottish National Blood Transfusion Service (SNBTS), Edinburgh, United Kingdom; 6 University of Edinburgh and SNBTS, Edinburgh, United Kingdom; University of Liverpool, United Kingdom

## Abstract

Variant CJD (vCJD) is an incurable, infectious human disease, likely arising from the consumption of BSE-contaminated meat products. Whilst the epidemic appears to be waning, there is much concern that vCJD infection may be perpetuated in humans by the transfusion of contaminated blood products. Since 2004, several cases of transfusion-associated vCJD transmission have been reported and linked to blood collected from pre-clinically affected donors. Using an animal model in which the disease manifested resembles that of humans affected with vCJD, we examined which blood components used in human medicine are likely to pose the greatest risk of transmitting vCJD via transfusion. We collected two full units of blood from BSE-infected donor animals during the pre-clinical phase of infection. Using methods employed by transfusion services we prepared red cell concentrates, plasma and platelets units (including leucoreduced equivalents). Following transfusion, we showed that all components contain sufficient levels of infectivity to cause disease following only a single transfusion and also that leucoreduction did not prevent disease transmission. These data suggest that all blood components are vectors for prion disease transmission, and highlight the importance of multiple control measures to minimise the risk of human to human transmission of vCJD by blood transfusion.

## Introduction

Human transmissible spongiform encephalopathies (TSEs), including vCJD, are characterised by vacuolar pathology in brain, alongside accumulation of a pathological form (PrP^Sc^) of the prion protein in neuronal and lymphoid tissues. Affected individuals usually exhibit a long period of asymptomatic infection followed by a shorter clinical phase. To date, there have been 174 cases of vCJD in the UK and a further 52 cases worldwide, the majority of which are thought to have been acquired directly from bovine sources [1], [2], [3]. As disease diagnosis is usually confirmed post mortem, typically by the detection of abnormal prion protein in neuronal or lymphoid tissues [4], [5], the prevalence of sub-clinical infection is of major concern and predicting the number of vCJD carriers is difficult. Current estimates suggest that between 1 in 4,000 to 1 in 10,000 people in the UK may be carriers of vCJD infection [6], [7], [8]. The methionine (M) – valine (V) polymorphism at codon 129 of the human prion protein gene (*PRNP*) is an important determinant of susceptibility to vCJD and other human TSEs. Although all clinical cases of vCJD have occurred in individuals homozygous for methionine at codon 129 (MM129), current clinical and experimental evidence indicates that individuals of all *PRNP* genotypes are susceptible to vCJD infection [9], [10]. The pre-clinical phase of disease in individuals with the MV129 or VV129 *PRNP* genotype is predicted to be longer than those with the MM genotype; moreover these individuals may not develop clinical disease but carry a subclinical infection and still have the potential to transmit infection. Hence there is obvious concern that human to human transmission from individuals sub-clinically infected with vCJD may amplify or prolong a vCJD outbreak in humans.

Of possible iatrogenic routes for human to human vCJD transmission (which include surgical and dental instruments), blood transfusion is of particular concern due to the widespread distribution of infectivity in lymphoid tissues observed in vCJD patients [11]. The potential sub-clinical prevalence of infection among donors, combined with infectivity titres in blood and the efficiency of blood as a route of transmission, are key factors which will influence the likelihood of acquiring vCJD by this route. To date, there have been four vCJD infections associated with the transfusion of non-leucoreduced red cell concentrates from donors who later developed vCJD [12], [13], [14]. The UK blood services have taken a number of measures to try to reduce the risk of transmission of vCJD by blood, plasma and tissue products, such as donor deferral, importation of plasma products and universal leucoreduction of components [15].

Estimating the risk of disease transmission has relied on rodent TSE models (infected intracerebrally [16], [17], [18], [19], [20], [21], [22]) to predict the distribution and titre of prion-associated infectivity in blood. Such experiments have suggested a titre of approximately10 infectious doses (ID)/ml in hamster blood [17], [23]. However, these models are not representative of the normal regimen of transfusion in clinical settings, as the volumes that were inoculated into brain were much lower than those that can be transfused by the intravenous route. Sheep orally infected with BSE provide an ideal model for studying transfusion-associated, blood-borne vCJD in that the distribution of PrP^Sc^ and infectivity in lymphoid tissues closely resembles that of vCJD patients [24]. Using this model, we were the first to show that prion diseases (scrapie and experimental BSE infection in sheep) could be transmitted via the transfusion of whole blood and buffy coat [25], [26], [27]. Subsequently, other large animal transfusion models have confirmed our findings [28], [29], [30]. A significant advantage of using the sheep-BSE transfusion model is that blood from sheep can be collected in similar volumes and processed to equivalent specifications as human blood components, using the same techniques employed in routine blood transfusion practice. We have further exploited this valuable model and undertaken a unique series of experiments to address the likelihood of transmission of vCJD following the transfusion of blood components used in clinical practice. Additionally we have assessed the efficacy of leucoreduction in either reducing or removing prion-associated infectivity in blood.

We report here, for the first time, several significant scientific advances using this model. All non-leucodepleted components (i.e. red cell concentrates, plasma units and platelet concentrates prepared to the specification used in a clinical setting) from BSE-infected animals are capable of causing prion disease in transfused recipients following a single transfusion. Transmission of disease occurred from blood collected early in the pre-clinical phase of disease. Moreover the process of leucofiltration of blood components did not prevent disease transmission.

## Methods

### Ethics statement

All animal work was reviewed and approved by ethical review panel at The Institute for Animal Health and The Roslin Institute and conducted under the authority of Home Office Project Licences (references:30/2282 and 60/4143 respectively).

### In vivo work

Some of the methods used in this study have been described elsewhere: genotyping; oral infection of donors; housing of sheep and observation of clinical signs [27]; cross matching of blood [25]; blood collection and transfusion of whole blood/components [26]. Sheep (sourced from the DEFRA scrapie-free flock) were all of the ARQ/ARQ PrP genotype (therefore susceptible to BSE infection [25], [31]) using the standard terminology for codons 136, 154 and 171. Individual animals differed in their genotype at other polymorphic codons of the PrP gene.

### Inoculation regime

**Figure 1:**
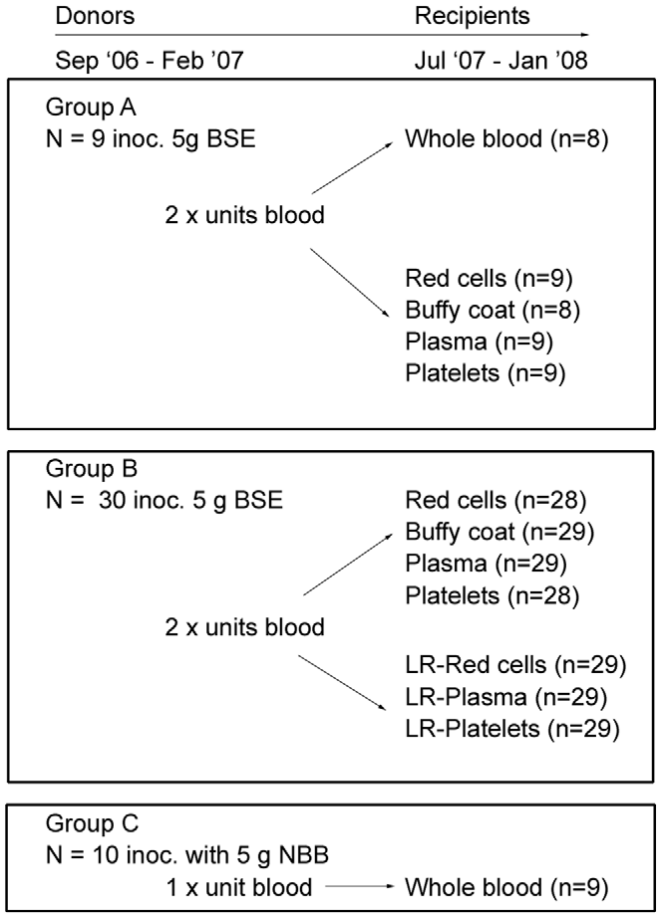
Overview of experimental design. Sheep used as BSE blood donors were divided into two experimental groups (A and B), though both groups were orally inoculated (inoc.) with 5 g of BSE brain homogenate at approximately 3–6 months of age. Two units of blood were collected from all BSE donors and processed according to the experimental regime described: group A sheep - one unit was transfused to recipients as whole blood, whereas blood from the second unit was processed into components and then transfused. Both units of blood from sheep in group B were separated into components and one set of components was passed through human leucoreduction (LR) filters prior to transfusion. Negative control donors (group C), were orally inoculated with normal bovine brain homogenate (NBB). A unit of whole blood was collected from these sheep and transfused to recipients.

To assess which clinically-relevant blood components have the potential to cause prion disease following transfusion, we first orally infected 40 TSE-free donor sheep (of the susceptible ARQ/ARQ PrP genotype) with 5 g of bovine BSE-infected brain homogenate. Groups of inoculations were performed at monthly intervals (typically eight sheep at a time) starting in September 2006 and continuing until February 2007. Of the 40 BSE-inoculated sheep, one died of intercurrent cause before blood could be collected for transfusion and therefore was excluded from the study. Mock-infected donors were inoculated with 5 g normal bovine brain homogenate at similar time intervals as the infected cohort (Figure 1).

### PrP^Sc^ detection

The preparation of brain/peripheral tissue homogenates for PrP^Sc^ detection (using Proteinase K (PK) digestion and sodium phosphotungstic acid (NaPTA) precipitation, gel electrophoresis and Western blotting with the monoclonal antibody BC6 at a concentration of 1 µg/ml) is described elsewhere [32]. A proportion of the PK and NaPTA-treated brain homogenates were deglycosylated using PNGaseF (New England Biolabs) using established methods [33] with a modification to the amount of enzyme added (typically 1500 U per reaction). Immunohistochemistry using the antibody BG4 was undertaken as previously described [25], with modification of the chromagen to Vector NovaRED (Vector Laboratories). Tissues from all sheep (regardless of whether they died following clinical signs of BSE/intercurrent causes or were culled for welfare reasons) were tested using these methods.

### Preparation of blood components for transfusion

Two units of whole blood (WB, 1 unit = 450 ml±10% (v/v)) were collected from each donor sheep (where possible) into quadruple, bottom and top (BAT) blood packs (Fresenius Hemocare, NPBI) fitted with inline leucoreduction filters, containing 63 ml of anti-coagulant CPD.A1 (citrate, phosphate, dextrose, adenine). Approximately 1 ml was sampled from the filled blood bags for analysis and then the majority of units (except eight units of whole blood) were centrifuged for 7.5 min, 1350 g at 20°C (Sorvall RC3BP, H6000A rotor). This resulted in three layers: the top layer was platelet-rich plasma (PRP), the interface contained buffy coat (BC) and the lower layer contained red cells. Each of these layers were extracted into empty, parallel blood packs using a Compomat G4 optimised for the separation of sheep blood components (Fresenius Hemocare, NPBI). Immediately after separation, 100 ml of the preservative SAG-M (saline, adenine, glucose and mannitol) was aseptically added to the red cells, as a replacement for the loss of plasma during separation, and thoroughly mixed. The PRP was centrifuged at 2350 g for 10 min at 22°C, yielding a platelet-poor plasma supernatant (the plasma unit) and a platelet-rich plasma pellet (the platelet concentrate). The plasma unit was extracted aseptically into a clean bag for transfusion, leaving the platelet concentrate (in a minimal volume of plasma). This was placed on a flat surface and ‘rested’ for 60 min at room temperature. The platelets were thoroughly resuspended by gentle mixing. The buffy coat fraction remained in the original collection bag. Red cells were leucoreduced (using the inline T3953 filter) after separation from whole blood. Platelet-rich plasma was first leucoreduced (using a leucoreduction filter from the Fresenius/NPBI CompoStop F730 system), then separated into plasma and platelets by centrifugation as described.

### Determining the volume of each component transfused

Prior to transfusion, 10–20 ml of each component were sampled for analysis and archive storage. After sampling was complete, the volume of each component to be transfused was calculated by subtracting the weight of an empty bag from the filled bag weight and adjusting for weight (g)/volume (ml) by dividing by the appropriate correction factor. We used specific gravities of 1.06 for whole blood, red cell concentrate and buffy coat, and 1.03 for plasma and platelets, as the correction factor for weight to volume determinations.

### Determining the distribution of plasma in components

The haematocrit (Hct) was used to determine the distribution (%) of plasma in the separated components. A sample of whole blood, red cells and buffy coat was drawn up into a plain glass capillary and then one end capped. The capillary was centrifuged for 6 min using a haematocrit centrifuge (Hawksley) and packed cell volume measured using a haematocrit reader (Hawksley). We used data provided by the Scottish National Blood Transfusion Service to estimate the total volume of plasma in whole blood, and then the percentage of the total plasma associated with red cell concentrate, buffy coat, plasma unit and platelet concentrate (and leucoreduced equivalents), following their preparation from whole blood [34].

### Leucocount analysis

Leucocytes in non-leucoreduced and leucoreduced blood components were measured using a Leucocount™ kit (BD Biosciences) in combination with flow cytometry. All leucodepleted samples were tested undiluted but non-leucodepleted blood samples were first diluted in PBS, pH 7.4: whole blood and red cell concentrates - 1∶ 500; buffy coat - 1∶1000; plasma was used neat and platelet concentrates diluted 1∶30. In brief, 100 µl of sample was added to TruCount tube which contained a known number of 4.2 µm fluorescent beads. To this, 400 µl of Leucocount™ reagent was added and samples were mixed and incubated in the dark for 5 min, at room temperature. The assay was validated using Leucocount™ combo quality control samples, which were treated in the same way as blood samples. A ‘blank’ was prepared by substituting 100 µl PBS, pH 7.4 in place of the blood sample (bead control). All samples were analysed using a 4-colour FACSCalibur (BD Biosciences). All test samples were analysed after assay parameters were satisfied and propidium iodide-stained cells were visualised in FL2 alone. From statistical analysis, region statistics were obtained and the leucocytes were enumerated using a simple calculation provided by the manufacturer.

### Platelet counting in plasma units and platelet concentrates

Given the size difference between sheep and human platelets, sheep samples could not be counted using a haematology analyser. Therefore, samples from the platelet concentrate and plasma units were first diluted (typically 1∶300 and 1∶7 respectively) in either BARRS fluid (0.25% (w/v) saponin, 3.5% (w/v) sodium citrate containing 1 ml of 40% (v/v) formaldehyde in a final volume of 100 ml distilled water, Sigma) or PBS, pH 7.4. Approximately 20 µl of diluted sample were used to fill a chamber of an improved Neubauer haemocytometer. Platelets were visualised using ×40 or ×100 objectives and residual platelet numbers were counted using standard techniques.

## Results

### BSE-infection status in animals used as blood donors

**Figure 2:**
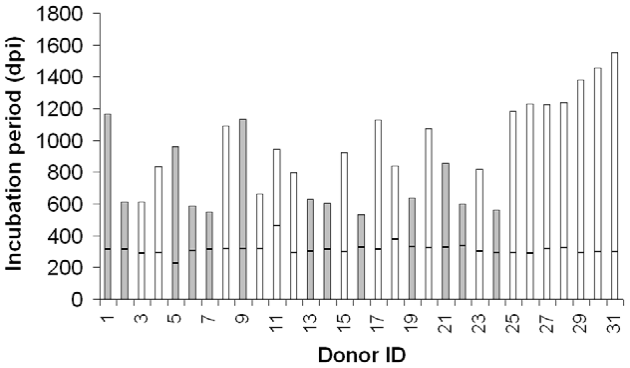
Varied incubation times are seen in blood donors following oral inoculation with BSE. All BSE-infected donor sheep (apart from donor 4, which was culled for welfare reasons) were culled on demonstration of clinical signs. The range of incubation period for sheep confirmed as having BSE is 534–1544 days post infection (dpi). The horizontal bar in each column represents the time of blood collection for separation and subsequent transfusion to recipients and ranges from 228–463 days post oral inoculation. To date, 31/39 donors have succumbed to BSE infection and the grey bars indicate donor blood, which when transfused, caused disease in recipients.

To date, 31 of the 39 infected donors have shown typical clinical signs and pathological evidence confirming BSE infection, with incubation periods ranging from approximately 534 to 1554 days (Figure 2). The incubation time may be influenced by a variety of factors including variability in uptake of the infectious agent following oral inoculation and the outbred genetic background of the sheep. We anticipate that some additional live BSE donors (n = 4) will develop disease, albeit with longer incubation periods. Four BSE challenged donors culled at 258, 265, 320 and 683 days post infection, were confirmed as negative for BSE (data not shown). One of ten mock-infected control donors died of intercurrent causes (266 days post-inoculation) and was confirmed negative for BSE infection. The survival period for the remaining donors (control and infected) is greater than 1500 days.

### Blood component preparation and specifications

The timing of blood collection from donors (in days post-inoculation) is indicated by the horizontal bar in each column in Figure 2. We aimed to prepare sheep blood components to the same specifications as those used for human blood [34], using similar processing methods and commercially available blood packs and leucoreduction filters. This produced components of whole blood (WB, n = 8), buffy coat (BC, n = 37) red cell concentrates (RCC, n = 37), plasma (PLS, n = 38), platelet concentrates (PLT, n = 37), leucoreduced red cell concentrates (LR-RCC, n = 29), leucoreduced plasma units (LR-PLS, n = 29) and leucoreduced platelet concentrates (LR-PLT, n = 29). The components were transfused into TSE-free recipients (ARQ/ARQ genotype, n = 244) over a periods of seven months. Nine units of whole blood were prepared from mock-infected donors as negative controls (Figure 1).

**Figure 3:**
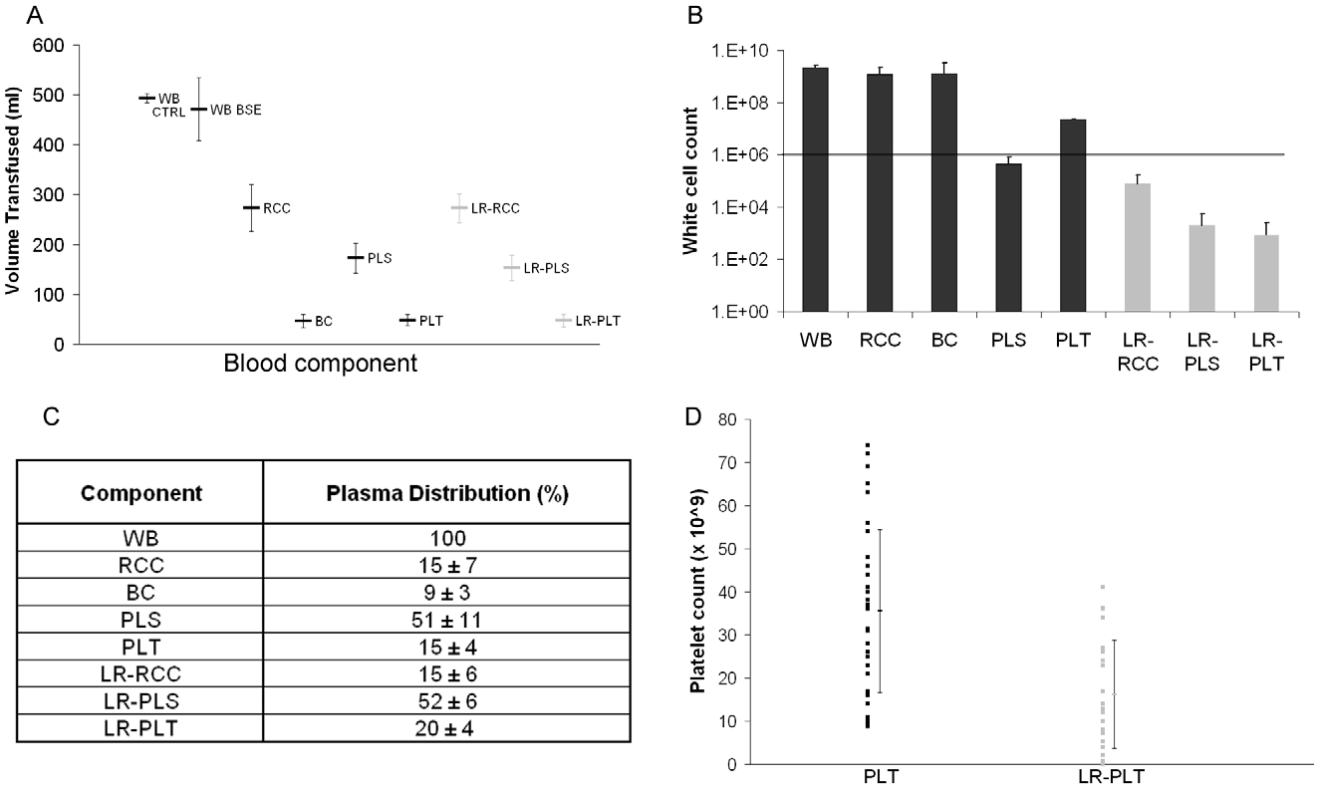
Analysis of sheep blood components. Graph A shows the range and average volume (±1SD) of each component used for transfusion. No difference was observed in the volume between leucoreduced (grey bars) and non-leucoreduced components (black bars). White cell counts were calculated using Leucocount™ kits and flow cytometry. All leucoreduced components from sheep (grey bars) meet the specifications assigned to human equivalents and contained less that 1×10^6^ residual leucocytes (as shown by the solid line, graph B). Following the separation of whole blood, the majority of the total plasma volume was distributed in association with plasma unit. The remaining plasma volume was differentially distributed in red cells, platelets and buffy coat, (panel C) as a percentage of the total plasma volume. No major differences were observed in plasma distribution between leucodepleted and non-leucodepleted components. The number of platelets in platelet concentrates prepared from leucoreduced and non-leucoreduced components was highly variable in sheep and the average count was statistically significantly different (p<1×10^−5^, using 2-tailed Student's t-test, graph D).

The majority of components prepared from sheep blood matched the specifications for the same components prepared from human blood. Similarities in the volumes of sheep and human components were observed (Table S1-A), and for sheep there was little change in component volume following leucoreduction (Figure 3A). In a clinical setting, leucoreduced components must contain less than 1×10^6^ residual leucocytes per unit. Figure 3B shows that LR-RCC, LR-PLS, LR-PLT, prepared from sheep, contained 7.65×10^4^, 12.02×10^3^, and 0.91×10^3^ average white cells/unit, respectively, and thus meet this criterion (Table S1-B, Figure S1). Interestingly, non-leucoreduced plasma also contained less than 1×10^6^ leucocytes per unit. Following centrifugation, the majority of ‘whole blood plasma’ formed the plasma unit, whilst the remainder differentially associated with red cells (∼15%), platelets (15–20%) and buffy coat (9%, Figure 3C). The number of platelets associated with both non- and leucoreduced plasma units contained less than <30×10^6^/ml and therefore met the specification required for the preparation of human plasma units (Table S1-C). The specification for platelet counts in human platelet concentrates was >60×10^9^ per unit. It can be seen that several equivalents prepared from sheep blood met this requirement, whilst others contained less than this (ranging from 9–74×10^9^). For leucoreduced platelet concentrates, the average platelet count was 16.23±12.59×10^9^ (ranging from 0.35–41×10^9^, from 29 individual preparations, Figure 3D). Platelets in all platelet concentrates were counted before and after leucoreduction and we noted that in sheep the platelet count was highly variable between animals, and that post-leucoreduction, platelet counts were consistently lower (Figure 3D). Furthermore, following extraction from platelet-rich plasma, aggregation of the platelets occurred in some units (as assessed using microscopy) which may have caused technical difficulties in counting, likely resulting in an under-estimate of platelet counts.

### All non-leucoreduced blood components can transmit disease

We have demonstrated that all blood components, used in routine transfusion medicine can support prion disease transmission by blood transfusion (Table 1).The volume transfused varied between components (from ∼50 ml volumes for buffy coat compared to ∼500 ml for whole blood units) and within components (i.e. platelet volumes ranged from 44 to 74 ml). It is important to remember that at the time of blood collection and donation, all donors were healthy and not exhibiting clinical signs of prion infection. Figure 2 highlights the donors from which positive transmissions of BSE have occurred (grey bars). It is conceivable that blood from some donors (though later confirmed as having BSE) did not contain sufficient infectivity titres at the time of donation to cause disease in transfused recipients. If this is the case, not all transfused recipients will succumb to infection. However, the positive transmissions observed here may provide a basis for understanding the mechanism of prion replication in blood, and subsequently the infection process. Our data also shows that the clinical end point for any single component transfused was different, which may be indicative of variable titres of infectivity from donors themselves, or reflect how infectivity is distributed amongst the separated components.

**Table 1:** Summary of BSE transmissions in recipients following blood transfusion.

Donors (D)			Recipients (R)	
ID	Component donated	Volume Transfused (ml)	ID	Cull (dpt)
D1	BC	92	R1-A*	631
D2	WB	358	R2-A	567
	RCC	300	R2-B†	616
	BC	25	R2-C	639
	PLS	185	R2-D	594
	PLT	46	R2-E	741
D5	PLT	74	R5-A	1001
D6	PLT	60	R6-A	701
	BC	44	R6-B	701
D7	WB	514	R7-A	792
	RCC	301	R7-B††	658
	BC	41	R7-D	904
	PLS	185	R7-E	953
	PLT	50	R7-C	609
D9	BC	46	R9-B	595
	PLT	44	R9-A	643
D13	RCC	307	R13-A	1008
	BC	45	R13-B	513
	PLS	164	R13-C	720
	PLT	60	R13-D†	391
	LR-RCC	324	R13-E	1008
	LR-PLS	121	R13-F	770
	LR-PLT	45	R13-G†	1120
D14	BC	63	R14-A	1146
D16	WB	555	R16-A	468
	RCC	287	R16-B	987
	BC	36	R16-C*	603
	PLS	173	R16-D	1089
	PLT	62	R16-E	974
D19	RCC	299	R19-B	841
	BC	28	R19-C	878
	PLT	45	R19-D	995
	LR-RCC	243	R19-A	681
D21	BC	55	R21-A	784
D22	RCC	269	R22-A	1063
	BC	43	R22-B	821
D24	RCC	254	R-24C	1105
	BC	52	R24-A	729
	PLS	203	R24-D*	652
	PLT	55	R24-B	1016

*indentifies animals that were culled because of inappetance, which we now recognise to be an early symptom of TSE infection occuring before overt clinical signs.

†indicates animals that were culled for other welfare, non-TSE causes.

††This animal died of intercurrent causes.

Table 1 shows details of blood components which, when transfused from donors to paired recipients, have resulted in BSE. The time point of euthanasia in recipients is indicated in days post transfusion (dpt) and animals were culled following presentation of clinical signs typical of BSE. In a limited number of cases, sheep were culled for welfare reasons. Transfused components are abbreviated as follows: whole blood (WB), red cell concentrates (RCC), buffy coat (BC), plasma (PLS), platelet concentrates (PLT) and leucoreduced equivalents are prefixed with ‘LR’. The volume of each component the recipients received is indicated in ml.

**Figure 4:**
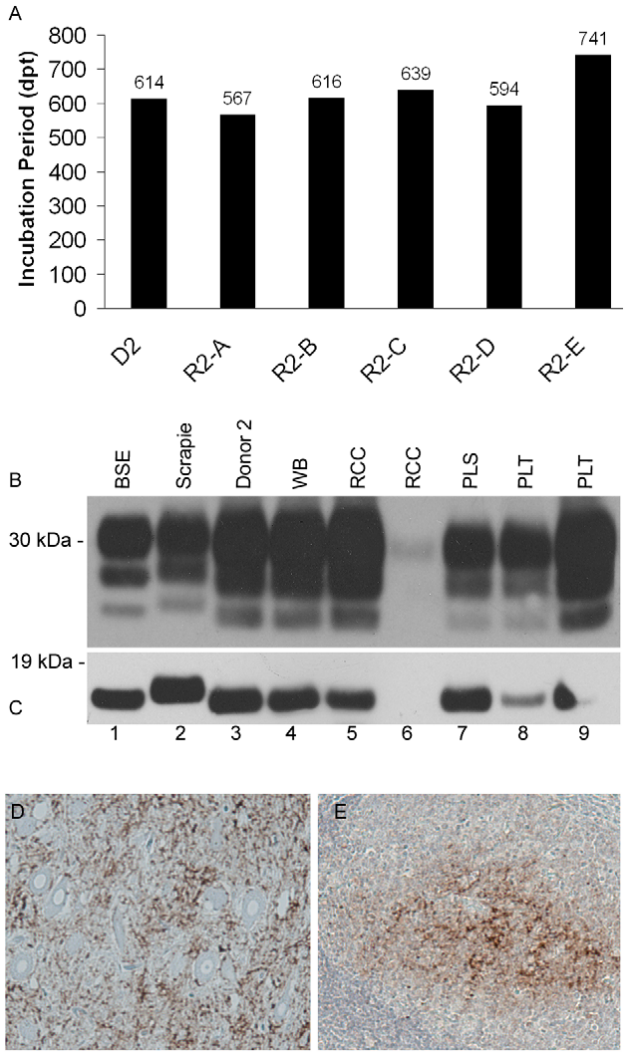
Confirmation of BSE in recipients of non-leucoreduced components. Panel A shows the incubation periods of donor 2 (D2) and its respective transfusion recipients of whole blood (R2-A), red cells (R2-B), buffy coat (R2-C), plasma (R2-D) and platelets (R2-E), whereby the nomenclature used to describe sheep is consistent with that in Table 1. Panel B shows PrP^Sc^ in brain from a donor and a range of transfusion recipients compared to PrP^Sc^ in experimental controls. Lane 1 – BSE control, lane 2 – sheep scrapie control, lane 3 – donor 2, lane 4 whole blood recipient, lanes 5 and 6 – two recipients of red cells, lane 7 – plasma recipient and lanes 8 & 9 – two recipients of platelets. Lane 6 shows detection of low levels of PrP^Sc^ in recipient 7B that received a unit of red cell concentrate and died of intercurrent causes before reaching clinical endpoints. The PrP^Sc^ profile is consistent with that of cattle BSE (lane 1) as expected. These data are confirmed following PNGase F treatment of PrP^Sc^ as shown in panel C. Panels D and E show abnormal prion protein deposition in dorsal motor nuclei of the vagus nerve (DMNV) in brain and tonsil of donor 16, respectively. Tissue sections were stained with BG4.

Following only a single blood transfusion to individual recipients, we have observed 36 positive transmissions of BSE following the transfusion of whole blood, red cell concentrate, buffy coat, plasma and platelet concentrate prepared from multiple donors. In some cases (Figure 4A) all non-leucoreduced components prepared from a single donor were sufficiently infectious to cause BSE in recipients (i.e. donor 2). Similar outcomes were observed when the same repertoire of components prepared from donors 7 and 16 were transfused to matched recipients (Table 1). In other examples, only some of the non-leucodepleted components transfused have resulted in disease in recipients (i.e. red cell concentrate and buffy coat from donor 22 or buffy coat and platelets from donor 9). To date, whole blood has shown the highest rate of transmission (37.5% of recipients infected), followed by buffy coat (32.4%), platelets (24.3%), then red cells (18.9%) and plasma (13.2%).

BSE was confirmed in recipients of non-leucodepleted components, post-mortem, by the detection of PK-resistant PrP^Sc^ (a marker for prion infection) by Western blotting assays or by antibody staining of abnormal prion protein in tissue sections. A range of tissues from each sheep were tested and included brain and peripheral lymphoid tissues (spleen, tonsil, pre-scapular and mesenteric lymph nodes). The PrP^Sc^ protein resolves, using SDS-PAGE, as three separate protein bands which correlate to different glycotypes. Figure 4B shows PrP^Sc^ in brain samples from recipients of infected whole blood, red cells, plasma and platelet concentrates (lanes 4, 5, 7, 8 and 9 respectively). The migration and protein profile of the disease-associated prion protein resembles that of cattle BSE as expected, (lane 1) and differs from that of sheep affected with natural scrapie (lane 2). This data is confirmed by further biochemical processing which removes the carbohydrates from the PrP^Sc^ protein, leaving only a single protein band (Figure 4C), whereby the BSE motif in transfused sheep becomes more obvious, resulting in the lower apparent molecular weight of the unglycosylated protein band compared to sheep scrapie. Brain and lymphoid follicles of the tonsil, from donor 16, also showed abnormal prion protein deposition, following antibody staining and immunohistochemical analysis (Figures 4D and E respectively). In a limited number of cases, sheep were culled before the onset of clinical signs and for other health reasons. This provided an opportunity to examine the distribution of PrP^Sc^ in different brain regions and peripheral tissues in sheep pre-clinically affected with BSE (Figures S2A–S2C).

### Leucoreduction of blood components does not prevent prion disease transmission via blood transfusion

**Figure 5:**
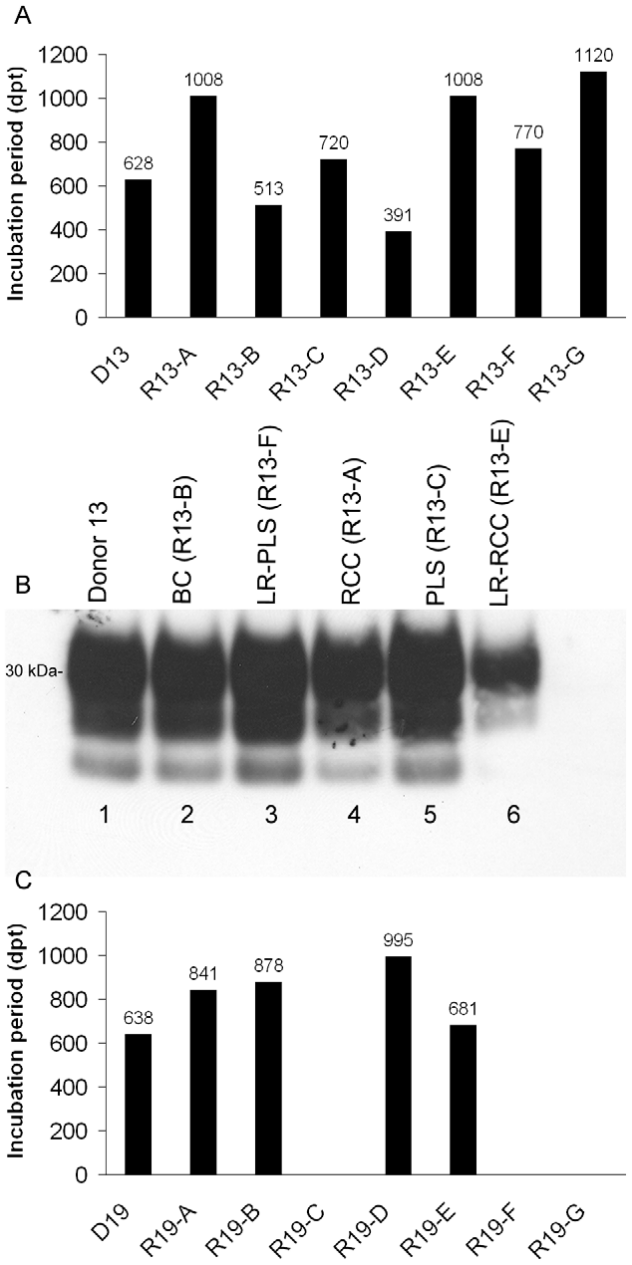
BSE is transmitted following the transfusion of leucoreduced components. Panel A shows the entire repertoire of components prepared from donor 13 (D13) and transfused. The corresponding incubation periods (defined as the date of death based on reaching defined clinical endpoints) is shown in days above the bars. Both platelet recipients (R13-D and R13-G) did not reach clinical endpoint, but were culled for welfare reasons (thus these ‘incubation periods’ correctly refer to the date of euthanasia, in days post transfusion (dpt)). Panel B is a representative immunoblot showing PrP^Sc^ in brain samples of donor 13 (lane 1) and selected, matched transfusion recipients of buffy coat, leucoreduced plasma, red cells, plasma and leucoreduced red cells (lanes 2–6 respectively), thus confirms BSE infection following the transfusion of leucoreduced blood components. Selected recipients of components, including leucoreduced red cells, from donor 19 were also confirmed to have BSE. All of these recipients developed a clinical disease at different time points post transfusion (panel C). Blood from donors 13 and 19 was collected at 48 and 52% of the final incubation period respectively; all later showed typical clinical signs of BSE and were confirmed as positive, as previously described.

Currently, we have observed transmissions of BSE following the transfusion of leucoreduced-red cells (Table 1; donors 13 and 19), -plasma and -platelets (donor 13). Figure 5A shows that all blood components prepared from donor 13 contained sufficient infectivity titres to cause BSE in transfused recipients. However, the striking observation was that even after leucoreduction, infectivity titres were still sufficient to transmit BSE. To date, in nearly all cases, little or no difference in the incubation period was seen in recipients of paired non-leucoreduced plasma and red cells and their leucoreduced equivalents (Figure 5A). This was mirrored in the comparable BSE-like protein profiles in brain of donor 13 and respective recipients' that received plasma, red cells and leucoreduced equivalents (Figure 5B). The same profile was detected in brain of recipient 13-G (not shown). However, for paired platelets and leucoreduced platelet recipients (13-D and 13-G respectively, Table 1), there is a difference of some 700 days in incubation time. Both of these recipients, whilst demonstrating intermittent, early clinical signs of BSE infection, were culled because of underlying welfare issues and so the true incubation period, i.e. the time between infection to clinical disease, are not accurately reflected by the cull dates. Indeed in the case of recipient 13-D (platelets), PrP^Sc^ was only detected in the spleen and pre-scapular lymph node, but not in brain (Figure S2-C) suggesting an early stage of infection and peripheral pathology.

Leucoreduced-red cell concentrate from donor 19 also transmitted BSE following transfusion to recipient 19-A (Figure 5C), though in an apparently shorter time frame than red cells alone. BSE was confirmed by the characteristic PrP^Sc^ profile in brain of donor 19 and its respective culled recipients (data not shown). Recipients that received leucoreduced-plasma and -platelets from donor 19 are still healthy and to date are not demonstrating clinical signs associated with BSE infection. In relation to the number of each type of leucoreduced component transfused, leucoreduced-red cells have shown the higher rate of transmission (6.9% of recipients infected), compared to 3.4% for both leucoreduced-plasma and -platelets (Table 2). However, these data represent interim transmission efficiencies and will likely change over the full time-course of the study. The survival period for the remaining recipients (control and infected) is greater than 1200 days

**Table 2:** Current transmission efficiencies of blood components.

	Challenge	No. animals inoculated	No. BSE +ve sheep	Transmission efficiency
Donors	5 g BSE brain	39	31	79%
Recipients	Whole blood	8	3	37.5%
	Red cells	37	7	18.9%
	Buffy coat	37	12	32.4%
	Plasma	38	5	13.2%
	Platelets	37	9	24.3%
	LR-Red cells	29	2	6.9%
	LR-Plasma	29	1	3.4%
	LR-Platelets	29	1	3.4%

Table 2 identifies the number of positive transmissions of BSE, by the component transfused to recipients. The current transmission efficiency of each type of blood component was determined by calculating the number of recipients confirmed as having BSE (post-mortem) as a percentage of the total number of each component transfused. The attack rate for orally infected BSE donor sheep is shown for comparison. Blood components prefixed by ‘LR’ mean that they were leucoreduced prior to transfusion.

### Prion infectivity in blood is present early in the pre-clinical phase of disease

The marked variability in incubation period observed in donor sheep following oral BSE infection was not anticipated (Figure 2). Given that we collected blood from donors around 200–400 days post-oral BSE infection, this provided an opportunity to examine the prion-associated infectivity in blood at different pre-clinical stages of disease. Extrapolation of our data shows that blood collected from confirmed BSE-infected donors at intervals between 27–61% of the incubation period is infectious (Table S2). For example, donor 5 was first inoculated with BSE (day 0); then 228 days later two units of blood were collected, processed and components transfused to recipients. This donor started to show clinical signs of BSE approximately 914 days after BSE infection and, following a short clinical phase, was euthanized. Prion-associated infectivity in this donor's blood was confirmed by transmission of BSE to transfused recipients, even though the donor was healthy and there was no clinical evidence to suggest prion disease at the time of blood donation. We calculated the timing of the blood donation and onset of clinical signs as a function of the final incubation period (i.e. date of euthanasia). We observed that infectivity in blood is present very early in the incubation period, with positive transmissions occurring from blood collected as early as 27% of the final incubation period (Table S2), which is earlier then previously reported [27], [35]. These are individual collections from each sheep, and not a true time course experiment. We cannot therefore conclude whether blood from donors 2, 7 and 16, collected at time points earlier than 51, 57 and 61% of the incubation period respectively, contained the same or different titres of infectivity and hence, if transfused, would result in BSE. It should also be noted that, in general, blood collected from the sheep with shorter incubation periods (<650 days, i.e. donors 2, 6, 7, 13, 14, 16, 19, 22 and 24) have transmitted BSE to a greater number of recipients than those donors with longer incubation periods (i.e. donors 1, 5, 9 and 21). This may reflect the levels of infectivity in the blood at the time of collection and donation [27].

## Discussion

We have shown that all of the blood components investigated, whole blood, plasma, red cells, platelets and buffy coat, are capable of transmitting BSE infection following transfusion into susceptible sheep. Although infectivity has consistently been detected in plasma in rodent scrapie models, this is the first time that transmission of prion disease has been demonstrated following transfusion of plasma units collected from pre-clinical donors, and prepared to the same specifications as non-fractionated human plasma. While the results with platelets and leucocytes are similar to that of Mathiason *et al.* [30], the plasma results are strikingly different. In the previous study, prion infected cell-free plasma appeared not to cause disease following transfusion in deer. The reason for this is not clear and may relate to either a difference in the distribution of PrP^Sc^ and/or infectivity in different host species or indeed the strain of infectious agent used.

We also observed a number of cases in which leucoreduced components have caused BSE in recipients following transfusion. Other recipients of leucoreduced components are also now showing early signs of TSE infection and we would therefore expect more positive transmissions of BSE from these components. These data demonstrate that leucoreduction of blood components alone is insufficient to prevent transmission of prion infection via blood transfusion [23]. All four of the documented transfusion-related transmissions of vCJD infectivity to date have occurred in individuals who received red cells (the most commonly transfused blood component) which were non-leucoreduced, between 1996 and 1999. Eighteen surviving recipients [36] transfused with red blood cells (n = 6), cryodepleted plasma (n = 1) and leucoreduced red blood cells (n = 11) from donors who were subsequently confirmed to have vCJD are still being followed up (personal communication from Miss J. McKenzie and Prof. R.G. Will, TMER study). There are a small number of cases of clinical vCJD with a history of blood transfusion, where no donor has developed clinical vCJD but where transfusion remains the possible source of infection. The most recent of these cases (2002) received leucoreduced red cells. Our findings would suggest that residual infectivity following leucoreduction may still pose a risk of transmission in a transfusion setting [23], [37], [38].

These transfusion studies were conducted in sheep, in which many effects of polymorphisms associated with the *prnp* gene (which modulates susceptibility to prion infection) are well understood. Similarly, the age at which donors and recipients were exposed to BSE prions, the infectious dose (in donor sheep) and the route of infection were relatively well controlled. Despite the control of known variables within this experiment, we observed significant variability in the incubation period of both orally infected donors and transfused recipients. During the course of this study we identified a novel effect of an existing polymorphism in the sheep *PRNP* gene, which modulates the incubation period of orally infected BSE donors (unpublished observations). While some of the variability in incubation periods of transfused recipients is likely associated with other genetic factors [39], [40], [41], it is also likely to be influenced by variability in prion titre in blood from donor sheep. It must also be recognised that in this model, incubation period is not considered an indicator of titre of infectivity in blood, since a full titration of infected blood has not yet been undertaken in sheep. However, given that BSE occurred in recipients following a single blood transfusion from donors who were healthy at the time of blood donation, we would suggest that even when or if the infectious load is low, disease can result if the route of transmission, i.e. blood transfusion, is highly efficient. Whilst we have clearly shown that blood collected from donor animals at a single time point is infectious, what remains unclear is when prion-associated infectivity first appears in blood, how it relates to incubation period and how the titre of infection changes as disease progresses. We are investigating these questions by inoculating transgenic mice with blood collected at various time points throughout the incubation period, from the same donor sheep as used in this study. This will allow us to address the duration, pattern and titre of prion infectivity in blood.

Our data raises considerable questions concerning the distribution of infectivity in blood, including its potential association with cell types other than leucocytes i.e. red cells and platelets, and/or other proteins or soluble components of plasma. Our data relating to the current transmission efficiencies of each component suggest whole blood and buffy coat represent the greatest risk in terms of transfusion and blood safety. However, these data may change over the full time course of this study and therefore it is too early to draw definitive conclusions. Furthermore, the blood components used in this study (including leucoreduced equivalents) were not purified cell populations, but also contained plasma and leucocytes to reflect the nature of the components routinely transfused in human patients. This may complicate understanding the process of identifying the relationship between the infectious agent and cell targets. It is also possible that mechanisms such as release of membrane fractions during processing [42] or shedding/transit by plasma membrane-derived microvesicles [43], [44] may also contribute to the dissemination of infectivity in blood. It will be critical to understand how prion infectivity associates with particular blood components, and may identify new targets for diagnostics, therapeutics and allow for more refined risk reduction strategies.

Whilst these findings highlight the difficulties in predicting incubation times and the clinical outcomes associated with transfusion-related transmission of vCJD in humans (because of the extensive variability of individuals in terms of age, genetic background, titre and route by which one could be exposed) these data have clear implications for transfusion practices. We demonstrate the potential risk of acquiring vCJD from any blood component used in routine transfusion medicine, following a single transfusion from asymptomatic individuals.

While there has been recent, significant, developments in the quest for a blood-based assay to detect prion infection in symptomatic individuals [45], implementing such assays in the blood transfusion service to detect asymptomatic donors requires extensive validation. Thus there currently remains the potential for human to human transmission of vCJD via blood transfusion from individuals carrying the infectious agent but not showing clinical signs of disease. Our data suggests that leucoreduction alone is inadequate to minimise the risk of transmission of vCJD and to ensure the safety of blood used in transfusions and ultimately to safeguard public health.

## Supporting Information

Figure S1 **Enumeration of leucocytes in transfused components using flow cytometry.** Leucocount reagent (BD Bioscience) was used to measure white cell counts in both leucoreduced and non-leucoreduced components, though non-leucoreduced components were first diluted before analysis. White cells are stained with propidium iodide (PI, shown in gate 2 in all dot plots). Absolute enumeration is achieved by comparing PI-cell staining against a known number of fluorescent beads (gate 1 in all dot plots) and sample volume assayed. The performance characteristic of the assay is calibrated against kit controls (panels B-E), including the bead control (panel A). Panels F, G and H show PI-stained leucocytes in red cell concentrates, plasma and platelet concentrates respectively. Following leucoreduction, little or no events were recorded in gate 2, indicating a gross reduction in the number of white cells contained in leucoreduced-red cells (panel I), plasma (panel J) and platelets (panel K). A simple calculation (provided by the manufacturers) is used to determine the leucocyte count, from those samples in which events in gate 2 were recorded.
(TIF)

Figure S2 **A: PrP^Sc^ distribution at the end stage disease.** At the clinical endpoint PrP^Sc^ is deposited in all brain regions and peripheral tissues from recipient 2-D, which received a unit of plasma from donor 2. Lane annotations are: 1 - Frontal cortex, 2 – Cerebellum, 3 – Thalamus, 4 – Midbrain, 5 – Pons, 6 – Medulla, 7 - Spinal cord, 8 – Spleen, 9 – Tonsil,10 - Distal ileal Peyers Patch, 11 - Mesenteric lymph node,12 - Pre-scapular lymph node. **B: PrP^Sc^ distribution in an animal that died from intercurrent causes.** Recipient 7-B (which received a unit of red cell concentrate from donor 7) died of intercurrent causes 658 days after transfusion and before demonstrating clinical signs of BSE infection. PrP^Sc^ was detected in the spleen of this recipient and lower levels in the pre-scapular lymph node. In brain, PrP^Sc^ was first detected in the medulla suggesting that PrP^Sc^ may be routed to the brain from the periphery, via the peripheral and autonomic nervous system, as reported by others [46], [47], [48], [49], [50]. Alternative routes of entry of BSE infection from blood directly into the brain have also been described [51], [52], [53]. Lane annotations are: 1 - Frontal cortex, 2 – Cerebellum, 3 – Thalamus, 4 – Midbrain, 5 – Pons, 6 – Medulla, 7 - Spinal cord, 8 – Spleen, 9 – Tonsil,10 - Distal ileal Peyers Patch, 11 - Mesenteric lymph node,12 - Pre-scapular lymph node. **C: PrP^Sc^ distribution in an animal culled for welfare reasons.** Recipient 13-D, received a unit of platelet concentrate from donor 13, and was culled on the basis of health concerns combined with early clinical signs, only 391 days after being transfused. In this case, the spleen and pre-scapular lymph node were the only lymphoid tissues which showed PrP^Sc^ reactivities. Previous studies have shown PrP^Sc^ deposition in tonsil following transfusion of BSE-infected blood [33], [54]. The differences observed in these studies are likely related to differences in the stage of infection at which the sheep were culled and tissues examined. Hence, our current data may suggest that tonsil is an unreliable indicator for early stages of TSE infection acquired by blood transfusion. PrP^Sc^ was not detected in the medulla (data not shown). Lane annotations are: 8 – Spleen, 9 – Tonsil,10 - Distal ileal Peyers Patch, 11 - Mesenteric lymph node,12 - Pre-scapular lymph node.
(TIF)

Table S1 **A: Volume of blood components transfused to sheep compared to human components.** Table S1-A shows that the average volume, (ml)±1SD, of each component prepared from BSE-infected sheep blood is within the normal specifications for the same components prepared from human blood. Whole blood (WB), red cell concentrate (RCC), plasma (PLS), platelets (PLT). Leucoreduced equivalents are pre-fixed with ‘LR’. *Note that buffy coat (BC) is not a product that is transfused (without further processing by the blood transfusions services). The volumes of platelets and plasma units used by the blood transfusion services are arbitrarily estimated, as the critical specifications for these components are the numbers of platelets that they contain as opposed to a specific volume prepared. The average volume for control whole blood transfused (n = 9) was 493 ml±9. **B: Leucocount analysis.** Table S1-B shows that all leucodepleted components used in our transfusion studies meet the same criteria as human equivalents and contain less than 1×10^6^ white blood cells per unit. The greatest concentration of white cells, following the separation of whole sheep blood, was associated with red cell concentrates and buffy coat, then distributed to a lesser extent within plasma and platelets. **C: Platelet analysis.** Table S1-C shows that the values obtained for platelet units were less than the specification recommended for human platelet units. This is likely due to platelets being trapped in the leucoreduction filters. It is important to note that sheep platelet units were not administered for medicinal purposes but were transfused in our study for qualitative comparisons of infectivity in blood components. The residual platelet concentration was, however, enriched 20 to 30-fold in leucoreduced and non-leucoreduced platelet concentrates compared to plasma unit equivalents respectively (data not shown).
(DOC)

Table S2 **Blood collection from donors as function of incubation time.** BSE infected donor sheep were first infected with 5 g BSE after which blood was collected. We aimed to collect blood for transfusion from donors about 50% (∼300–350 days) of the way through the final incubation period (based on estimates of clinical disease occurring between 20–22 months (600–720 days) post oral infection, hence when sheep would still be healthy and asymptomatic of BSE infection. We have observed positive transmissions of BSE from donors 1, 2, 5, 6, 7, 9, 13, 16, 19, 21 and 24 (bold and underlined). From this, we have observed for the first time using this model, that blood for BSE infected donors contains significant levels of infectivity from as early as 24% of the way through the final incubation time (donor 5) to 61% of the final incubation period (donor 16).
(DOC)
