# Supplementary figures and images for: All Clinically-Relevant Blood Components Transmit Prion Disease following a Single Blood Transfusion: A Sheep Model of vCJD
Source: PLoS One. 2011 Aug 17;6(8):e23169. doi: 10.1371/journal.pone.0023169 (PMC3157369; doi:10.1371/journal.pone.0023169)

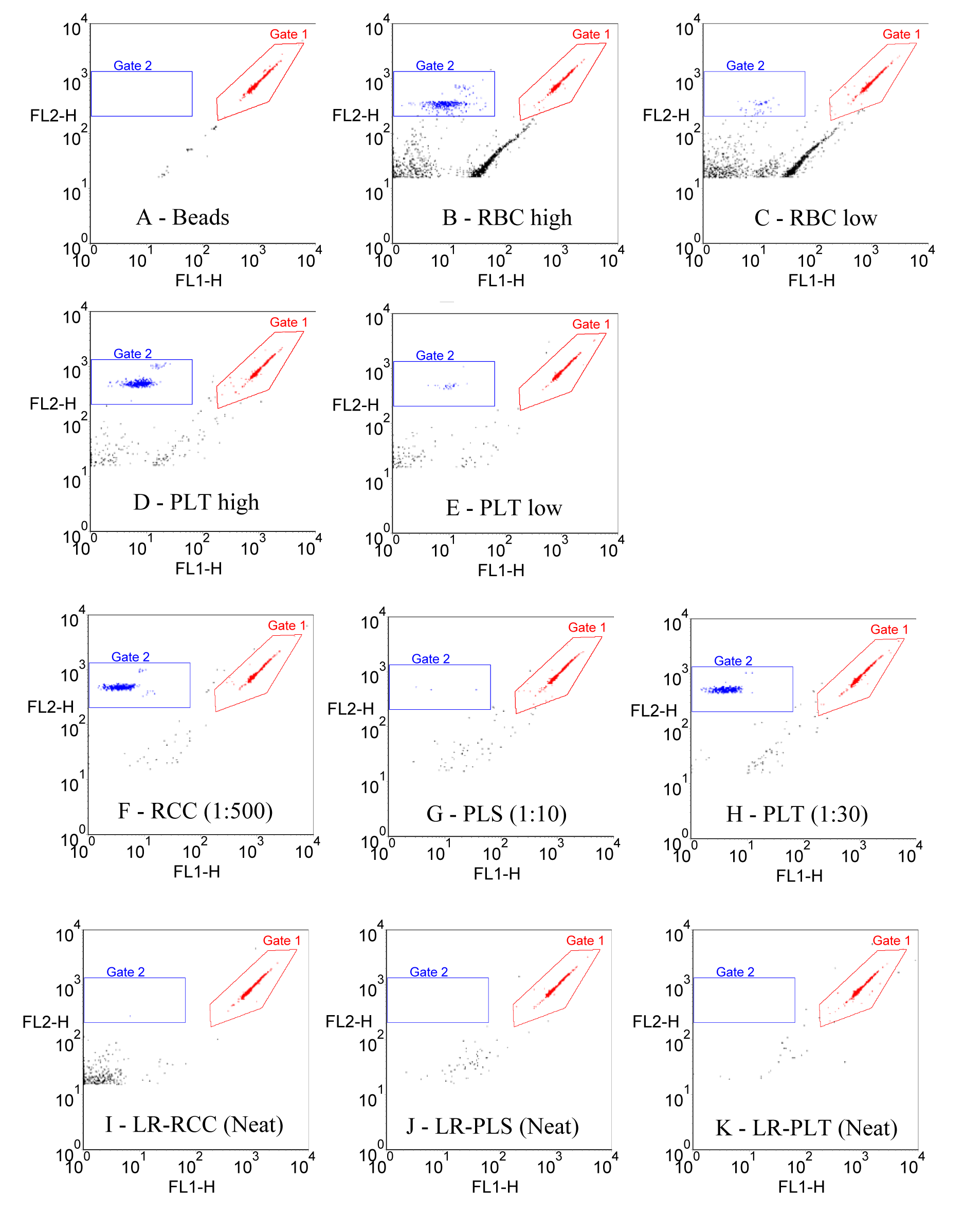

Supplement: Figure S1 — Enumeration of leucocytes in transfused components using flow cytometry. Leucocount reagent (BD Bioscience) was used to measure white cell counts in both leucoreduced and non-leucoreduced components, though non-leucoreduced components were first diluted before analysis. White cells are stained with propidium iodide (PI, shown in gate 2 in all dot plots). Absolute enumeration is achieved by comparing PI-cell staining against a known number of fluorescent beads (gate 1 in all dot plots) and sample volume assayed. The performance characteristic of the assay is calibrated against kit controls (panels B-E), including the bead control (panel A). Panels F, G and H show PI-stained leucocytes in red cell concentrates, plasma and platelet concentrates respectively. Following leucoreduction, little or no events were recorded in gate 2, indicating a gross reduction in the number of white cells contained in leucoreduced-red cells (panel I), plasma (panel J) and platelets (panel K). A simple calculation (provided by the manufacturers) is used to determine the leucocyte count, from those samples in which events in gate 2 were recorded. (TIF) [file pone.0023169.s001.tif]

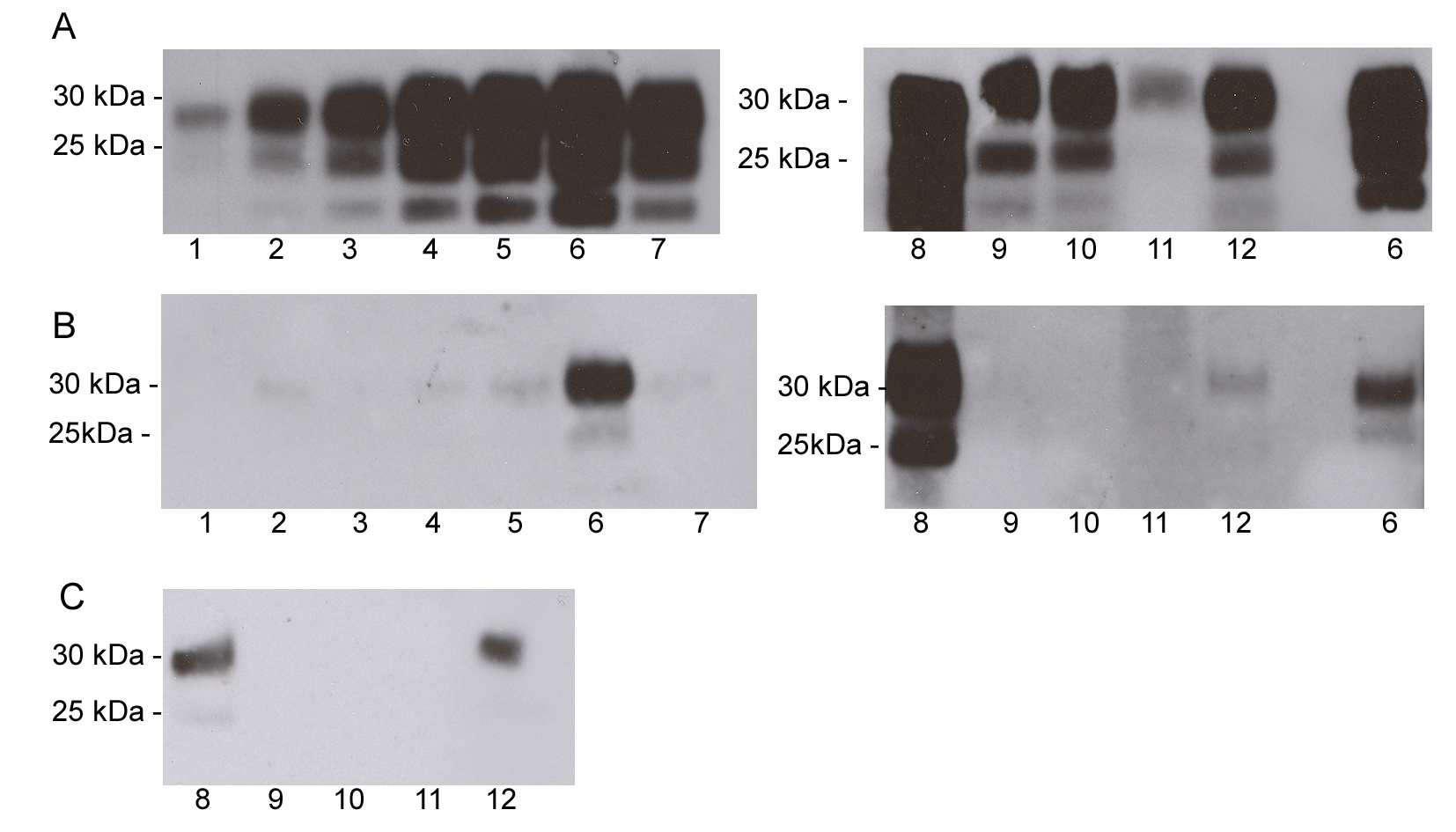

Supplement: Figure S2 — A: PrPSc distribution at the end stage disease. At the clinical endpoint PrPSc is deposited in all brain regions and peripheral tissues from recipient 2-D, which received a unit of plasma from donor 2. Lane annotations are: 1 - Frontal cortex, 2 – Cerebellum, 3 – Thalamus, 4 – Midbrain, 5 – Pons, 6 – Medulla, 7 - Spinal cord, 8 – Spleen, 9 – Tonsil,10 - Distal ileal Peyers Patch, 11 - Mesenteric lymph node,12 - Pre-scapular lymph node. B: PrPSc distribution in an animal that died from intercurrent causes. Recipient 7-B (which received a unit of red cell concentrate from donor 7) died of intercurrent causes 658 days after transfusion and before demonstrating clinical signs of BSE infection. PrPSc was detected in the spleen of this recipient and lower levels in the pre-scapular lymph node. In brain, PrPSc was first detected in the medulla suggesting that PrPSc may be routed to the brain from the periphery, via the peripheral and autonomic nervous system, as reported by others [46], [47], [48], [49], [50]. Alternative routes of entry of BSE infection from blood directly into the brain have also been described [51], [52], [53]. Lane annotations are: 1 - Frontal cortex, 2 – Cerebellum, 3 – Thalamus, 4 – Midbrain, 5 – Pons, 6 – Medulla, 7 - Spinal cord, 8 – Spleen, 9 – Tonsil,10 - Distal ileal Peyers Patch, 11 - Mesenteric lymph node,12 - Pre-scapular lymph node. C: PrPSc distribution in an animal culled for welfare reasons. Recipient 13-D, received a unit of platelet concentrate from donor 13, and was culled on the basis of health concerns combined with early clinical signs, only 391 days after being transfused. In this case, the spleen and pre-scapular lymph node were the only lymphoid tissues which showed PrPSc reactivities. Previous studies have shown PrPSc deposition in tonsil following transfusion of BSE-infected blood [33], [54]. The differences observed in these studies are likely related to differences in the stage of infection at which the sheep were culled and [file pone.0023169.s002.tif]
