# Supplementary material for: All Clinically-Relevant Blood Components Transmit Prion Disease following a Single Blood Transfusion: A Sheep Model of vCJD
Source: PLoS One. 2011 Aug 17;6(8):e23169. doi: 10.1371/journal.pone.0023169 (PMC3157369; doi:10.1371/journal.pone.0023169)
Supplement: Table S1 — A: Volume of blood components transfused to sheep compared to human components. Table S1-A shows that the average volume, (ml)±1SD, of each component prepared from BSE-infected sheep blood is within the normal specifications for the same components prepared from human blood. Whole blood (WB), red cell concentrate (RCC), plasma (PLS), platelets (PLT). Leucoreduced equivalents are pre-fixed with ‘LR’. *Note that buffy coat (BC) is not a product that is transfused (without further processing by the blood transfusions services). The volumes of platelets and plasma units used by the blood transfusion services are arbitrarily estimated, as the critical specifications for these components are the numbers of platelets that they contain as opposed to a specific volume prepared. The average volume for control whole blood transfused (n = 9) was 493 ml±9. B: Leucocount analysis. Table S1-B shows that all leucodepleted components used in our transfusion studies meet the same criteria as human equivalents and contain less than 1×10^6^ white blood cells per unit. The greatest concentration of white cells, following the separation of whole sheep blood, was associated with red cell concentrates and buffy coat, then distributed to a lesser extent within plasma and platelets. C: Platelet analysis. Table S1-C shows that the values obtained for platelet units were less than the specification recommended for human platelet units. This is likely due to platelets being trapped in the leucoreduction filters. It is important to note that sheep platelet units were not administered for medicinal purposes but were transfused in our study for qualitative comparisons of infectivity in blood components. The residual platelet concentration was, however, enriched 20 to 30-fold in leucoreduced and non-leucoreduced platelet concentrates compared to plasma unit equivalents respectively (data not shown). (DOC) [file pone.0023169.s003.doc]

| **Component** | **Volume (ml)** | |
| --- | --- | --- |
|  | **Sheep** | **Human** |
| WB | 471 ± 63 | 468 - 558 |
| RCC | 273 ± 46 | 220 - 340 |
| BC* | 46 ± 13 | NA |
| PLS | 173 ± 30 | 150 - 170 |
| PLT | 49 ± 11 | ~ 50 |
| LR-RCC | 273 ± 29 | 220 - 340 |
| LR-PLS | 153 ± 26 | 150 - 170 |
| LR-PLT | 48 ± 13 | ~ 50 |

**Supplementary Tables S1 A-C**

**A: Volume of blood components transfused to sheep compared to human components.**

**B: Leucocount analysis.**

| **Component** | **White cell count per unit** | |
| --- | --- | --- |
|  | **Sheep** | **Human** |
| WB | 2.16 x 10^9^ ± 0.45 x 10^9^ | NA |
| RCC | 1.22 x 10^9^ ± 1.02 x 10^9^ | NA |
| BC | 1.30 x 10^9^ ± 2.05 x 10^9^ | NA |
| PLS | 4.56 x 10^5^ ± 3.28 x 10^5^ | NA |
| PLT | 2.23 x 10^7^ ± 1.55 x 10^7^ | NA |
| LR-RCC | 7.65 x 10^4^ ± 9.01 x 10^4^ | < 1 x 10^6^ / unit |
| LR-PLS | 2.02 x 10^3^ ± 3.43 x 10^3^ | < 1 x 10^6^ / unit |
| LR-PLT | 0.91 x 10^3^ ± 1.63 x 10^3^ | < 1 x 10^6^ / unit |

**C: Platelet analysis.**

| **Component** | **Average platelet count in**  **Sheep blood** | **Specifications for human**  **blood** |
| --- | --- | --- |
| PLT | 36 x 10^9^ | > 60 x 10^9^ |
| LR-PLT | 17 x 10^9^ | > 60 x 10^9^ |
| PLS | 6.76 x 10^6^ / ml | < 30 x 10^6^ / ml |
| LR-PLS | 5.83 x 10^6^ / ml | < 30 x 10^6^ / ml |
