# Supplementary material for: All Clinically-Relevant Blood Components Transmit Prion Disease following a Single Blood Transfusion: A Sheep Model of vCJD
Source: PLoS One. 2011 Aug 17;6(8):e23169. doi: 10.1371/journal.pone.0023169 (PMC3157369; doi:10.1371/journal.pone.0023169)
Supplement: Table S2 — Blood collection from donors as function of incubation time. BSE infected donor sheep were first infected with 5 g BSE after which blood was collected. We aimed to collect blood for transfusion from donors about 50% (∼300–350 days) of the way through the final incubation period (based on estimates of clinical disease occurring between 20–22 months (600–720 days) post oral infection, hence when sheep would still be healthy and asymptomatic of BSE infection. We have observed positive transmissions of BSE from donors 1, 2, 5, 6, 7, 9, 13, 16, 19, 21 and 24 (bold and underlined). From this, we have observed for the first time using this model, that blood for BSE infected donors contains significant levels of infectivity from as early as 24% of the way through the final incubation time (donor 5) to 61% of the final incubation period (donor 16). (DOC) [file pone.0023169.s004.doc]

**Supplementary Table S2: Blood collection from donors as function of incubation time.**

| **Donor** | **Incubation**  **Period (IP, dpi)** | **Blood collected**  **From donors (dpi)** | **Blood collection as**  **A function of IP (%)** |
| --- | --- | --- | --- |
| **1** | **1168** | **315** | **27** |
| **2** | **614** | **313** | **51** |
| 3 | 614 | 289 | 47 |
| 4 | 835 | 292 | 35 |
| **5** | **959** | **228** | **31** |
| **6** | **586** | **305** | **52** |
| **7** | **551** | **314** | **57** |
| 8 | 1092 | 317 | 29 |
| **9** | **1132** | **317** | **28** |
| 10 | 664 | 319 | 48 |
| 11 | 944 | 463 | 49 |
| 12 | 797 | 295 | 37 |
| **13** | **628** | **301** | **48** |
| **14** | **603** | **314** | **52** |
| 15 | 924 | 296 | 32 |
| **16** | **534** | **326** | **61** |
| 17 | 1128 | 315 | 28 |
| 18 | 840 | 378 | 45 |
| **19** | **638** | **332** | **52** |
| 20 | 1076 | 323 | 30 |
| **21** | **858** | **326** | **38** |
| **22** | **602** | **33** | **56** |
| 23 | 817 | 302 | 37 |
| **24** | **564** | **293** | **52** |
| 25 | 1185 | 295 | 25 |
| 26 | 1231 | 291 | 24 |
| 27 | 1225 | 317 | 26 |
| 28 | 1239 | 324 | 26 |
| 29 | 1380 | 294 | 21 |
| 30 | 1455 | 299 | 21 |
| 31 | 1544 | 299 | 19 |
